# Identification of a Circulating Amino Acid Signature in Frail Older Persons with Type 2 Diabetes Mellitus: Results from the Metabofrail Study

**DOI:** 10.3390/nu12010199

**Published:** 2020-01-12

**Authors:** Riccardo Calvani, Leocadio Rodriguez-Mañas, Anna Picca, Federico Marini, Alessandra Biancolillo, Olga Laosa, Laura Pedraza, Jacopo Gervasoni, Aniello Primiano, Giorgia Conta, Isabelle Bourdel-Marchasson, Sophie C. Regueme, Roberto Bernabei, Emanuele Marzetti, Alan J. Sinclair, Giovanni Gambassi, on behalf of the European MID-Frail Consortium

**Affiliations:** 1 Università Cattolica del Sacro Cuore, 00168 Rome, Italy; riccardo.calvani@gmail.com (R.C.); anna.picca1@gmail.com (A.P.); jacopo.gervasoni@policlinicogemelli.it (J.G.); anielloprim@gmail.com (A.P.); Roberto.Bernabei@unicatt.it (R.B.); giovanni.gambassi@unicatt.it (G.G.); 2 Fondazione Policlinico Universitario “Agostino Gemelli” IRCCS, 00168 Rome, Italy; 3 Servicio de Geriatría, Hospital Universitario de Getafe, 28905 Madrid, Spain; leocadio.rodriguez@salud.madrid.org; 4 Department of Chemistry, Sapienza Università di Roma, 00185 Rome, Italy; federico.marini@uniroma1.it (F.M.); giorgia.conta@uniroma1.it (G.C.); 5 Department of Physical and Chemical Sciences, Università degli Studi dell’Aquila, 67100 L’Aquila, Italy; alessandra.biancolillo@univaq.it; 6 Foundation for Biomedical Research, Hospital Universitario de Getafe, 28905 Madrid, Spain; olga.laosa@salud.madrid.org (O.L.); laura.pedraza@salud.madrid.org (L.P.); 7 Centre Hospitalier Universitaire de Bordeaux, 33000 Bordeaux, France; isabelle.bourdel-marchasson@chu-bordeaux.fr (I.B.-M.); sophie.regueme@chu-bordeaux.fr (S.C.R.); 8 Foundation for Diabetes Research in Older People, Diabetes Frail Ltd., Luton LU1 3UA, UK; sinclair.5@btinternet.com

**Keywords:** aging, metabolomics, systems biology, personalized medicine, metabolism, frailty, sarcopenia, muscle wasting, precision medicine, metabolic profiling

## Abstract

Diabetes and frailty are highly prevalent conditions that impact the health status of older adults. Perturbations in protein/amino acid metabolism are associated with both functional impairment and type 2 diabetes mellitus (T2DM). In the present study, we compared the concentrations of a panel of circulating 37 amino acids and derivatives between frail/pre-frail older adults with T2DM and robust non-diabetic controls. Sixty-six functionally impaired older persons aged 70+ with T2DM and 30 age and sex-matched controls were included in the analysis. We applied a partial least squares-discriminant analysis (PLS-DA)-based analytical strategy to characterize the metabotype of study participants. The optimal complexity of the PLS-DA model was found to be two latent variables. The proportion of correct classification was 94.1 ± 1.9% for frail/pre-frail persons with T2DM and 100% for control participants. Functionally impaired older persons with T2DM showed higher levels of 3-methyl histidine, alanine, arginine, glutamic acid, ethanolamine sarcosine, and tryptophan. Control participants had higher levels of ornithine and taurine. These findings indicate that a specific profile of amino acids and derivatives characterizes pre-frail/frail older persons with T2DM. The dissection of these pathways may provide novel insights into the metabolic perturbations involved in the disabling cascade in older persons with T2DM.

## 1. Introduction

Type 2 diabetes mellitus (T2DM) is a chronic condition frequently occurring in old age [1,2]. T2DM is associated with higher risk of negative outcomes, including disability and mortality [3]. T2DM-related complications are especially prevalent in older adults and account for the increasing costs of T2DM [4]. Frailty defines a geriatric syndrome characterized by reduced ability to cope with life stressors and increased risk of adverse events (e.g., falls, delirium, loss of independence, mortality) [5,6]. T2DM and frailty are intimately related and share common features, including complex pathophysiology and heterogeneous phenotypes [7], that challenge their management [5,8].

Muscle failure, both in its metabolic and functional manifestations, is a hallmark of T2DM and frailty [7,9]. The progressive and generalized loss of muscle mass, strength, and function with age, termed sarcopenia, fuels a self-reinforcing cycle in which structural, metabolic, and endocrine perturbations in muscle exacerbate T2DM-related signs and symptoms [10]. T2DM further promotes the decline in muscle mass and function [11,12] which, in turn, aggravates functional impairment [13].

The central role of muscle wasting in frailty and T2DM may guide the identification of novel biomarkers and possibly new treatment targets for the two conditions [14,15,16]. In this context, circulating amino acids are promising candidates given their sensor-transducer-effector role in systemic metabolism, muscle homeostasis, and physical function [17,18,19,20]. Moreover, amino acids are involved in processes critical to the development and progression of frailty and T2DM, such as inflammation, glucose homeostasis, and redox regulation [21,22,23].

Targeted metabolomics allowed identifying specific amino acid profiles that were associated with insulin resistance and risk of developing T2DM in independent cohorts across US, Europe, and China [24,25,26,27]. A plasma amino acid signature, together with specific circulating lipid species, was linked to glucose dyshomeostasis and impaired insulin sensitivity in older adults from the Baltimore Longitudinal Study of Aging (BLSA) [28]. In addition, distinct patterns of circulating amino acids were associated with frailty and/or muscle-related parameters (mass, turnover, performance) in older individuals at risk for frailty [29,30,31,32]. Finally, within the “BIOmarkers associated with Sarcopenia and PHysical frailty in EldeRly pErsons” (BIOSPHERE) study, a combination of serum amino acids and derivatives was identified that characterized the metabotype of older adults with physical frailty and sarcopenia (PF&S) [17].

Here, we sought to define the circulating amino acid profile of frail/pre-frail older adults with T2DM (F-T2DM). Our approach, described in the context of the “Metabolic biomarkers of frailty in older people with type 2 diabetes mellitus” (MetaboFrail) study, coupled targeted metabolomics with a chemometric modeling strategy [16]. Through this innovative biomarker discovery strategy, we identified a specific profile of serum amino acids in F-T2DM older people. These findings may offer new insights into the metabolic perturbations associated with the disabling cascade in older persons with T2DM.

## 2. Materials and Methods

### 2.1. Study Population

MetaboFrail was developed as an ancillary study of the “Multi-modal Intervention in Diabetes in Frailty” (MID-Frail) project [16,33,34]. The latter was a cluster-randomized multicenter clinical trial that evaluated the effectiveness of a multicomponent intervention (mainly based on resistance exercise and lifestyle counseling) on improving physical performance compared with usual care in F-T2DM older adults from seven European countries (ClinicalTrials.gov identifier: NCT01654341) [16,33]. For the present study, a subgroup of MID-Frail participants recruited in Spanish and French study centers were enrolled. The main eligibility criteria were: (a) age at screening 70 years or older; (b) T2DM diagnosis from at least two years; and (c) being pre-frail or frail according to Fried’s criteria [35]. The main exclusion criteria were: (a) poor cognition operationalized as a Mini Mental State Examination score <20 [36]; (b) severe disability defined as a Barthel index score <60 [37]; critical conditions and/or major illnesses with a life expectancy <6 months; (c) inability or unwillingness to provide informed consent. Control participants were enrolled at the Università Cattolica del Sacro Cuore (Rome, Italy) and had the following characteristics: 70+ years of age, no T2DM, and no functional impairment. The study protocol was approved by local ethics committees according to both national and international laws. Prior to enrolment, all participants provided written informed consent. The study was conducted in agreement with legal requirements and international norms (Declaration of Helsinki, 1964).

### 2.2. Blood Collection and Determination of Serum Concentrations of Amino Acids and Derivatives

Blood samples were collected after overnight fasting. For serum separation, blood samples were kept on ice for about 30 min until clotting and were subsequently centrifuged at 1000× *g* for 10 min at 4 °C. Serum samples were eventually aliquoted and stored at −80 °C until analysis.

Concentrations of 37 amino acids and derivatives were determined in serum by ultraperformance liquid chromatography/mass spectrometry (UPLC/MS), as described previously [17]. Briefly, 50 μL of sample was added to 100 μL 10% (w/v) sulfosalicylic acid containing an internal standard mix (50 μM; Cambridge Isotope Laboratories, Inc., Tewksbury, MA, USA) and centrifuged at 1000× *g* for 15 min. Ten μL of the resulting supernatant were mixed with 70 μL of borate buffer and 20 μL of AccQ Tag reagents (Waters Corporation, Milford, MA, USA) and heated at 55 °C for 10 min. Samples were eventually loaded onto a CORTECS UPLC C18 column 1.6 μm 2.1 × 150 mm (Waters Corporation) for chromatographic separation (ACQUITY H-Class, Waters Corporation, Milford, MA, USA). Elution was performed at 500 μL/min flow rate with a linear gradient (9 min) from 99:1 to 1:99 water 0.1% formic acid/acetonitrile 0.1% formic acid. Analytes were detected on an ACQUITY QDa single quadrupole mass spectrometer equipped with electrospray source operating in positive mode (Waters Corporation, Milford, MA, USA). Amino acid controls (MCA laboratory of the Queen Beatrix Hospital, Winterswijk, The Netherlands) were used to monitor the analytic process.

### 2.3. Statistical Analysis

The normal distribution of data was ascertained through the Kolmogorov-Smirnov test. Comparisons between F-T2DM and control participants for normally distributed continuous variables were performed by *t*-test statistics. The non-parametric test Mann-Whitney U was applied to assess differences for non-normally distributed continuous data. Differences in categorical variables between groups were determined via χ^2^ statistics. Descriptive analyses were performed using the GraphPrism 5.03 software (GraphPad Software, Inc., San Diego, CA), with statistical significance set at *p* < 0.05.

### 2.4. Partial Least Squares-Discriminant Analysis and Double Cross-Validation Procedures

In order to unveil possible differences in circulating amino acid patterns between F-T2DM and control participants, a multivariate classification strategy based on partial least squares-discriminant analysis (PLS-DA) modeling was adopted [38]. PLS-DA is a classification method particularly suited for dealing with highly correlated predictors, as it is based on projecting the predictors (measured variables) onto a reduced subspace of latent variables (LVs; directions in space) of highest covariance with the responses, i.e., providing the maximum separation between classes. In order to validate the results of PLS-DA modeling and rule out the possibility that good results were obtained because of chance correlation, a procedure based on repeated double cross-validation (DCV) and permutation tests was used [39,40]. DCV consists of spitting the samples to obtain two cross-validation loops, an internal loop for model building/model selection and an outer loop that mimics external (test set) validation. The DCV procedure is repeated a sufficient number of times such that estimates do to depend on one specific sample splitting. This allows evaluating the consistency of model parameters and the confidence intervals for model predictions. To assess the statistical significance of the obtained predictions, the figures of merit which summarize the classification accuracy in repeated DCV [i.e., number of misclassifications (NMC), area under the receiver operating characteristic curve (AUROC), and discriminant (DQ2)] are compared with their distribution under the null hypothesis, which is estimated non-parametrically through permutation tests with 1000 randomizations. A more detailed description of the procedure can be found elsewhere [41]. PLS-DA and DCV were run under Matlab R2015b environment by means of in-house written functions (freely downloadable at: https://www.chem.uniroma1.it/romechemometrics/research/algorithms/plsda/).

## 3. Results

### 3.1. Study Population

The present investigation included 66 F-T2DM older adults and 30 age and sex-matched robust, non-diabetic controls. The main characteristics of the two groups are reported in Table 1. F-T2DM and control participants were comparable for age, sex distribution, and number of diseases. F-T2DM older adults showed higher body mass index relative to controls. As expected, a significant difference was found in physical functional between groups, as indicated by the scores on the short physical performance battery (SPPB).

### 3.2. Identification of Circulating Amino Acid Profiles

In the present study, we aimed at identifying profiles of circulating amino acids that discriminate older persons with F-T2DM from functionally intact non-diabetic peers. Among the available statistical options, we selected a PLS-DA-based strategy for its ability to handle multiple interdependent variables. The best PLS-DA model was built using two LVs. As indicated by the stringent DCV applied, the classification performance of the model was almost perfect. Indeed, the proportion of correct classification of participants was 96.6 ± 1.5% over the calibration sets (95.0 ± 2.2% for cases and 100.0 ± 0.0% for controls), 96.6 ± 1.5% (95.0 ± 2.2% for cases and 100.0 ± 0.0% for controls) in the internal DCV loop (i.e., the one used for model selection), and 95.9 ± 1.3% (94.1 ± 1.9% for cases and 100.0 ± 0.0% for controls) in the outer DCV loop, which accounts for the results of repeated external validation.

**Figure 1:**
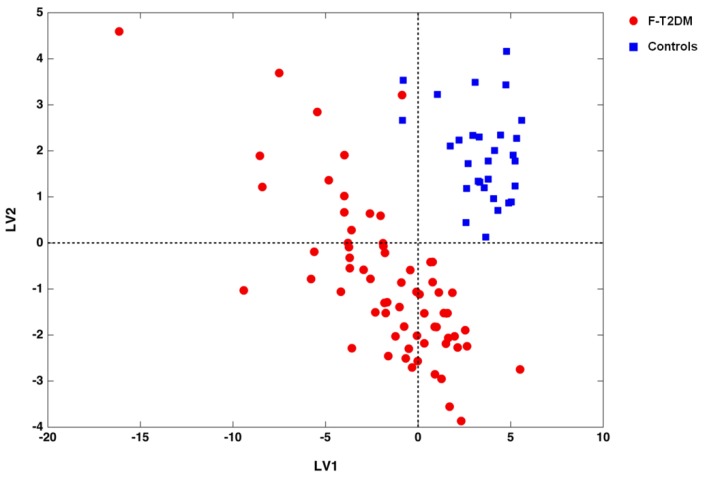
Scores plot showing the separation of frail/pre-frail older adults with type 2 diabetes mellitus (F-T2DM) from control participants according to the serum concentrations of amino acids and derivatives in the space spanned by the two latent variables (LV1 and LV2), as determined by partial least squares-discriminant analysis.

The remarkable classification performance of the PLS-DA model can be appreciated by inspecting the projection of study participants over the space spanned by the two LVs (Figure 1).

**Figure 2:**
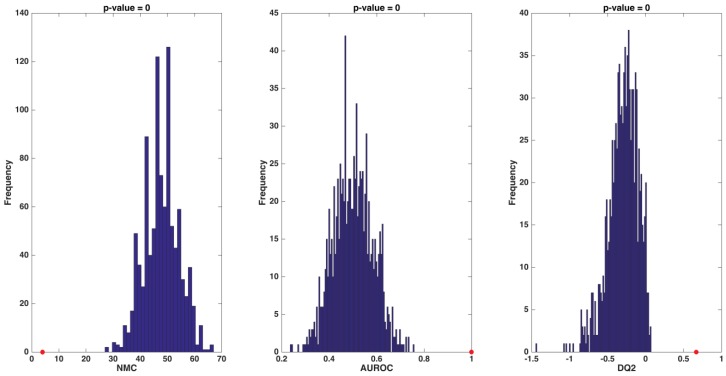
Distribution of values of number of misclassifications (NMC), area under the receiver operating characteristic curve (AUROC), and discriminant Q2 (DQ2) under their respective null hypothesis as estimated by permutation tests (blue histograms) and the corresponding values obtained by the partial least squares-discriminant analysis model on unpermuted data (red circles). Values obtained on the real dataset (red circles) fall outside of the corresponding null hypothesis distribution (blue histograms), corresponding to a *p* < 0.05.

A sharp separation between F-T2DM participants and controls is evident. To ensure the reliability of our findings against the possibility of chance correlations, DCV results of the PLS-DA model were compared with the distributions of specific figures of merit under the null hypothesis. As depicted in Figure 2, for all of the figures considered (i.e., NMC, AUROC, and DQ2), values obtained from the unpermuted dataset fell outside the corresponding null hypothesis distribution, indicating a *p* value <0.05.

The identification of the metabolites with the greatest discriminating power was accomplished by inspecting variable importance in projection (VIP) indices. Table 2 reports variables with a VIP value higher than 1.

F-T2DM participants showed higher serum levels of 3-methyl histidine, alanine, arginine, ethanolamine, glutamic acid, sarcosine, and tryptophan. Instead, controls were characterized by higher circulating levels of ornithine and taurine. Serum concentrations of non-discriminant analytes in the two participant groups are listed in Table S1.

## 4. Discussion

Over the last few years, analytical platforms have been developed together with sophisticated computational algorithms to allow the study of complex diseases in unprecedented detail [42]. This new healthcare paradigm, called personalized or precision medicine, incorporates “omics” technologies to unveil the inner biological properties of morbid conditions [42] and devise innovative diagnostics and interventions tailored to the needs of single individuals [42,43]. Metabolomics, by virtue of its privileged position at the interface between biological pathways and clinical manifestations of healthy/disease conditions [44], are improving our understanding of “normal” physiology and the pathophysiology of many disorders, including frailty and T2DM [15,45,46]. These premises led to the design of the MetaboFrail study [16].

In the present investigation, we applied targeted metabolomics to characterize the circulating amino acid profile of functionally impaired older persons with T2DM. Our major finding was that a specific pattern of amino acids discriminated F-T2DM older people from age- and sex-matched controls. The amino acid signature of F-T2DM participants included higher circulating levels of 3-methyl histidine, alanine, arginine, ethanolamine, glutamic acid, sarcosine, and tryptophan.

The presence of 3-methyl histidine among the most relevant predictors supports the involvement of muscle wasting in frailty [30]. Indeed, 3-methyl histidine derives from the post-translational methylation of histidine moieties of actin and myosin [47,48]. Hence, 3-methyl histidine has been proposed as a biomarker of myofibrillar proteolysis and skeletal muscle loss [49]. Interestingly, 3-methyl histidine is also a marker of increased protein catabolism in T2DM [50].

Alanine and glutamic acid are crucial intermediates of muscle energy metabolism and liver-muscle metabolic interchange under both physiologic and pathologic conditions [51,52,53]. Perturbations in alanine and glutamate circulating pool may be indicative of skeletal muscle dysfunction, and are commonly encountered in age-related chronic conditions and models of muscle atrophy [54,55]. Notably, both alanine and glutamic acid levels were positively associated with insulin resistance and risk of T2DM in several independent study cohorts [56,57,58].

Arginine metabolism involves the cooperation of various organs, including kidneys, muscles, gut, and liver [59]. Major pathways in arginine metabolism include muscle protein breakdown and its de novo synthesis from citrulline [59]. Arginine is involved in nitric oxide as well as urea and polyamine synthesis, and research focus has recently been directed towards the determination of the pathophysiological role of arginine and its metabolites in aging and age-related chronic diseases [60]. Noticeably, our findings mirror the higher levels of arginine and lower concentrations of its urea-cycle companion ornithine found in Chinese adults with T2DM [61]. Higher concentrations of another intermediate of urea-cycle, i.e., citrulline, defined the serum amino acid profile of older people with PF&S [17]. Further investigations on arginine/nitrogen interorgan networks are needed to explain the results found in F-T2DM older adults.

Sarcosine, the N-methylated derivative of glycine, is an important intermediate of one-carbon metabolism [62]. Recent studies have investigated the association between sarcosine levels and age-related conditions in humans with conflicting results [63,64,65,66]. In a comparative metabolomics analysis, circulating sarcosine was found to be reduced with aging and increased by dietary restriction in both rodents and humans [67]. Conversely, elevated urine sarcosine levels were associated with incident T2DM [68], and higher serum concentrations of sarcosine characterized the metabotype of older adults with PF&S [17]. Collectively, these findings suggest that perturbations in folate/one-carbon metabolism may play a role in frailty and T2DM as well as in other age-related conditions.

Ethanolamine is a crucial intermediate of the CDP-ethanolamine pathway, the main route of phosphatidylethanolamine synthesis, and modulates lipid metabolism and the turnover of biological membranes [69]. Recently, a critical role for skeletal muscle phospholipid metabolism has been described in the regulation of both insulin sensitivity and contractile function [70]. Moreover, the ethanolamine/phosphatidylethanolamine synthesis dyad is directly involved in autophagy regulation, thereby modulating anti-aging properties of this cellular process [71]. Interestingly, perturbations in the CDP-ethanolamine pathway were associated with altered mitochondrial biogenesis in mouse models of muscle atrophy [71], while higher circulating levels of ethanolamine were found in older adults with PF&S [17].

Tryptophan is an essential amino acid that exerts multiple roles in growth, mood, behavior, and immune responses [72]. Tryptophan is metabolized via two major pathways, the tryptophan-kynurenine and the tryptophan-methoxyndole pathways, that lead to the production of NAD, serotonin, and melatonin [72]. Alterations in tryptophan metabolism have been described in the context of frailty and T2DM [73,74]. In particular, tryptophan and its associated metabolites have been associated with insulin resistance and incident T2DM in independent cohorts [74,75]. Moreover, higher circulating levels of tryptophan were associated with low muscle quality in a large cohort of men and women enrolled in the BLSA [32].

Non-frail non-T2DM controls were characterized by higher serum levels of taurine. Taurine is the most abundant free amino acid in several organs, including the skeletal muscle that contains approximately 70% of total body taurine [76]. Taurine has multiple regulatory effects and its role in osmoregulation, modulation of inflammatory response, protection against oxidative stress, and stimulation of cellular quality control processes is widely acknowledged [76,77]. Taurine deficiency is commonly encountered in people with T2DM [78,79], and its supplementation has been proposed as a strategy against T2DM complications, including retinopathy, nephropathy, neuropathy, atherosclerosis, and cardiomyopathy [80]. Recently, taurine supplementation has also been proposed as a possible remedy against sarcopenia [81]. The causes of taurine depletion in F-T2DM older adults are multifaceted and may include decreased dietary intake, reduced intestinal absorption, renal wasting, and inflammation [77].

Unexpectedly, branched-chain amino acids (BCAAs) were not found to be discriminant by the PLS-DA classification model. BCAAs are metabolic rheostats that modulate whole-body and tissular metabolism [20]. Recent evidence suggests that BCAAs may have a Janus-like behavior in F-T2DM older adults. Indeed, while BCAA-induced activation of the mammalian target of rapamycin (mTOR) in skeletal myocytes may contrast sarcopenia and functional decline in advanced age [20,31], other studies found an association between higher circulating levels of BCAAs and insulin resistance or T2DM in older adults [24,25,26,27]. The low discriminant power of BCAAs in MetaboFrail might therefore reflect the dual effect of BCAAs in F-T2DM older people.

Although innovative, the present investigation has some limitations that should be mentioned. The study sample was quite small while the dataset comprised numerous variables. To cope with this issue, we adopted a PLS-DA-based strategy which is particularly suited for handling matrices populated by highly correlated variables. The MetaboFrail study enrolled older adults from three European countries; thus, a validation study in other ethnic groups is warranted. Eating habits may affect circulating amino acid levels, but diet was not objectively assessed in our study sample. However, it has recently been reported that differences in blood amino acid concentrations do not necessarily mirror those of amino acid intakes [82]. As commonly occurs in biomarker discovery studies, although a large number of candidates were investigated, we could not evaluate all possible mediators involved in frailty and T2DM. 

In conclusion, in the present investigation, we showed the existence of a specific amino acid signature in F-T2DM older persons. Our novel approach enabled us to obtain new insights into the pathophysiology of the two conditions. In particular, a relevant role for perturbations in muscle metabolism and muscle-liver interorgan communication was highlighted. The longitudinal implementation of our analytical strategy could allow validating novel sets of biomarkers and identifying new targets for interventions.

## Figures and Tables

**Table 1:** Main characteristics of study participants.

	F-T2DM (*n* = 66)	Controls (*n* = 30)	*p*
Age, years (mean ± SD)	76.5 ± 14.5	74.6 ± 4.3	0.46
Sex (female), *n* (%)	32 (48)	16 (53)	0.82
BMI, kg/m^2^ (mean ± SD)	29.2 ± 4.9	26.7 ± 2.4	0.01
SPPB score (mean ± SD)	8.6 ± 2.9	11.3 ± 0.9	<0.0001
Number of diseases (mean ± SD) ^§^	2.8 ± 1.0	2.9 ± 2.0	0.86

^§^ Includes hypertension, coronary artery disease, prior stroke, peripheral vascular disease, diabetes, chronic obstructive pulmonary disease, and osteoarthritis; Abbreviations: BMI, body mass index; F-T2DM, frail/pre-frail older adults with type 2 diabetes mellitus; SD, standard deviation; SPPB, short physical performance battery.

**Table 2:** Serum concentrations of discriminant biomolecules as resulted from partial least squares-discriminant analysis.

Analytes	F-T2DM (*n* = 66)	Controls (*n* = 30)
3-methylhistidine	7.8 ± 4.2	5.2 ± 2.5
Alanine	542.3 ± 165.8	384.3 ± 98.3
Arginine	168.3 ± 91.2	103.7 ± 31.2
Ethanolamine	11.5 ± 3.4	9.0 ± 2.2
Glutamic acid	130.0 ± 66.8	54.3 ± 21.3
Ornithine	103.2 ± 37.6	109.4 ± 25.0
Sarcosine	2.5 ± 0.9	1.6 ± 0.6
Taurine	100.4 ± 49.0	189.5 ± 47.2
Tryptophan	66.2 ± 23.4	62.0 ± 13.1

Data are shown as mean ± standard deviation. Concentrations are expressed in µmol/L.

## Supplementary Materials

The following are available online at https://www.mdpi.com/2072-6643/12/1/199/s1; Table S1: Serum concentrations of non-discriminant amino acids and derivatives in frail/pre-frail participants with type 2 diabetes mellitus (F-T2DM) and controls.
