# Supplementary material for: Identification of a Circulating Amino Acid Signature in Frail Older Persons with Type 2 Diabetes Mellitus: Results from the Metabofrail Study
Source: Nutrients. 2020 Jan 12;12(1):199. doi: 10.3390/nu12010199 (PMC7019630; doi:10.3390/nu12010199)
Supplement: Supplementary file 1 [file nutrients-12-00199-s001.pdf]

Table S1. Serum concentrations of non-discriminant amino acids and derivatives in frail/pre-frail participants with type 2 diabetes mellitus (F-T2DM) and controls.

Analytes	F-T2DM (n = 66)	Controls (n = 30)
α-amino butyric acid	29.5 ± 12.9	22.3 ± 5.7
β-alanine	8.5 ± 3.3	6.5 ± 2.6
β-aminobutyric acid	2.9 ± 1.5	1.7 ± 1.1
1-methylhistidine	10.6 ± 10.1	9.2 ± 16.7
3-methylhistidine	7.8 ± 4.2	5.2 ± 2.5
4-hydroxyproline	17.6 ± 8.8	17.2 ± 7.9
Alanine	542.3 ± 165.8	384.2 ± 98.3
Aminoadipic acid	2.7 ± 1.7	1.3 ± 0.8
Arginine	168.3 ± 91.2	103.7 ± 31.2
Asparagine	78.8 ± 20.0	77.8 ± 13.4
Aspartic acid	22.0 ± 8.2	17.0 ± 4.0
Citrulline	36.5 ± 14.8	36.8 ± 11.5
Cystine	5.2 ± 4.7	32.4 ± 11.3
Ethanolamine	11.5 ± 3.4	9.0 ± 2.2
Glycine	253.0 ± 73.0	260.9 ± 65.6
Glutamic acid	130.0 ± 66.7	54.3 ± 21.3
Histidine	95.3 ± 24.0	82.2 ± 11.4
Isoleucine	91.9 ± 31.8	63.2 ± 18.3
Leucine	170.2 ± 55.4	121.9 ± 27.0
Lysine	251.8 ± 85.9	196.8 ± 41.6
Methionine	28.2 ± 9.8	23.4 ± 5.8
Ornithine	103.2 ± 37.6	109.4 ± 25.0
Phenylalanine	85.7 ± 28.6	66.1 ± 11.0
Proline	268.2 ± 100.4	199.9 ± 44.4
Sarcosine	2.5 ± 0.9	1.5 ± 0.5
Serine	140.1 ± 41.1	118.7 ± 16.9
Taurine	100.4 ± 49.0	189.5 ± 47.2
Threonine	137.9 ± 51.5	125.1 ± 22.3
Tryptophan	66.2 ± 23.4	62.0 ± 13.1
Tyrosine	78.2 ± 25.3	65.7 ± 14.6
Valine	302.7 ± 90.3	221.2 ± 42.9

Data are shown as mean ± standard deviation. Concentrations are expressed in μmol/L.
Concentrations of γ-aminobutyric acid, anserine, carnosine, cystathionine, phosphoethanolamine, and phosphoserine were below the detection limit.
